# Comparing weight loss rates between males and females: a preliminary analysis

**DOI:** 10.1080/15502783.2025.2550187

**Published:** 2025-08-23

**Authors:** Valentina Rodriguez Da Silva, Ryan Singh, Valeria V. Silva Ramirez, Daniel Arevalo, Eric T. Trexler, Landon Shannahan, John Solis, Samuel Blanke, Samuel Evers, Talon Westervelt, John Holtje, Bill I. Campbell

**Affiliations:** a University of South Florida, Exercise Science & Kinesiology Program, Performance & Physique Enhancement Lab, Tampa, FL, USA; b Duke University, Department of Evolutionary Anthropology, Durham, NC, USA

**Keywords:** Caloric deficit, body mass, gender difference, fat loss

## Abstract

**Background:**

Biological differences between males and females include variations in fat-free mass, resting metabolic rate, and hormone profiles. Males have more lean muscle and visceral fat, likely associated with differences in testosterone, while females have higher body fat and subcutaneous fat, likely associated with differences in estrogen and progesterone. Given the differences in physiological profiles, it could be hypothesized that males and females respond differentially to fat loss efforts. This study investigated the difference in the time it takes for males and females to lose 5% of their body weight during a uniformly prescribed caloric deficit.

**Methods:**

Resistance-trained participants [323 total, 268 females (83%), 55 males (17%)] underwent a 30% caloric deficit from their maintenance intake and were allocated up to 15 weeks to lose 5% of their bodyweight. Protein (1.6 g/kg) and calories were prescribed, and participants logged their intake using MyFitnessPal. After the diet was completed, 95 participants [78 females (82%), 17 males (18%)] were able to finish it in its entirety. Males and females time to achieve ≥5% of body mass loss were analyzed first by using Levene’s test for homogeneity of variance between groups, and then using Welch’s two-sample t-test to compare the number of weeks required to lose ≥5% of body mass between genders.

**Results:**

After the 15-week period, a total of 78 out of 268 females (29.1%) and 17 out of 55 males (30.9%) achieved a ≥5% reduction in body weight. There were no differences between biological sexes in homogeneity of variance between groups (*p* = 0.2819). Additionally, there was no significant difference in the number of weeks required to lose at least 5% of body mass between males and females (*p* = 0.3492). Mean time to reach this threshold was 9.45 ± 3.38 weeks for females and 8.71 ± 2.80 weeks for males.

**Conclusion:**

Although males and females present different biological profiles that result in differing body composition outcomes, under a controlled caloric deficit, males and females lose 5% of body weight at similar rates, with no significant difference between groups. These findings imply that individual variability likely plays a greater role in weight loss than biological sex. Future research should explore the factors contributing to this variability and include more balanced samples for validation.

